# Resistance to TKIs in EGFR-Mutated Non-Small Cell Lung Cancer: From Mechanisms to New Therapeutic Strategies

**DOI:** 10.3390/cancers14143337

**Published:** 2022-07-08

**Authors:** Andreas Koulouris, Christos Tsagkaris, Anna Chiara Corriero, Giulio Metro, Giannis Mountzios

**Affiliations:** 1 Thoracic Oncology Center, Theme Cancer, Karolinska University Hospital, 17177 Stockholm, Sweden; andreas.koulouris@gmail.com; 2 Faculty of Medicine, University of Crete, 70013 Heraklion, Greece; chriss20x@gmail.com; 3 School of Medicine, Faculty of Health, Education, Medicine & Social Care, Anglia Ruskin University, Bishop Hall Lane, Chelmsford CM1 1SQ, UK; annacorrero@gmail.com; 4 Giulio Metro, Medical Oncology, Santa Maria della Misericordia Hospital, Azienda Ospedaliera di Perugia, 06132 Perugia, Italy; giulio.metro@ospedale.perugia.it; 5 Clinical Trials Unit, Fourth Department of Medical Oncology, Henry Dunant Hospital Center, 11526 Athens, Greece

**Keywords:** EGFR, TKIs, non-small cell lung cancer, resistance, osimertinib, immunotherapy

## Abstract

**Simple Summary:**

Resistance to tyrosine kinase inhibitors (TKIs) of the epidermal growth factor receptor (EGFR) in advanced mutant non-small cell lung cancer (NSCLC) constitutes a therapeutic challenge. Resistance may occur as a result of EGFR-dependent and independent molecular pathways. The first commonly includes T790M, C797S, L792X and L718X mutations, while the latter pertains to HER2 and MET amplifications, gene rearrangements, disruption in PIK3CA, MAPK signaling and SCLC and epithelial–mesenchymal cells transformation. Liquid biopsies detecting mutant cell-free DNA (cfDNA) have a major potential in the detection of mutant clones before they become clinically apparent. Newer-generation TKIs, bispecific antibodies and antibody-drug conjugates or combinations of TKIs with other TKIs or chemotherapy, immunotherapy and anti-vascular endothelial growth factors (anti-VEGFs) are currently in use or under investigation in EGFR mutant NSCLC. In EGFR mutant NSCLC metastatic to the brain, the blood–brain barrier (BBB) decreases the ability of TKIs to reach the central nervous system (CNS), acting as an additional resistance factor, which can presently be addressed with osimertinib. The potential of rechallenging EFGR TKIs after chemotherapy and combining it with anti-PD-1 immunotherapeutics remains ambivalent. Harnessing nanocarriers to improve drug delivery in EGFR TKIs-resistant NSCLC has been promising in preclinical settings, but it is yet to be determined in a clinical context.

**Abstract:**

Resistance to tyrosine kinase inhibitors (TKIs) of the epidermal growth factor receptor (EGFR) in advanced mutant Non-Small Cell Lung Cancer (NSCLC) constitutes a therapeutic challenge. This review intends to summarize the existing knowledge about the mechanisms of resistance to TKIs in the context of EGFR mutant NSCLC and discuss its clinical and therapeutic implications. EGFR-dependent and independent molecular pathways have the potential to overcome or circumvent the activity of EGFR-targeted agents including the third-generation TKI, osimertinib, negatively impacting clinical outcomes. CNS metastases occur frequently in patients on EGFR-TKIs, due to the inability of first and second-generation agents to overcome both the BBB and the acquired resistance of cancer cells in the CNS. Newer-generation TKIs, TKIs targeting EGFR-independent resistance mechanisms, bispecific antibodies and antibody-drug conjugates or combinations of TKIs with other TKIs or chemotherapy, immunotherapy and Anti-Vascular Endothelial Growth Factors (anti-VEGFs) are currently in use or under investigation in EGFR mutant NSCLC. Liquid biopsies detecting mutant cell-free DNA (cfDNA) provide a window of opportunity to attack mutant clones before they become clinically apparent. Overall, EGFR TKIs-resistant NSCLC constitutes a multifaceted therapeutic challenge. Mapping its underlying mutational landscape, accelerating the detection of resistance mechanisms and diversifying treatment strategies are essential for the management of the disease.

## 1. Introduction

According to data by the World Health Organization, lung cancer was the most common cause of cancer-related death worldwide in 2020, with 1.80 million deaths (WHO, 2021). Non-small-cell lung cancer (NSCLC), which accounts for approximately 85% of lung cancer diagnoses globally, has been classified by WHO as a heterogenous group comprising mainly adenocarcinomas and squamous cell carcinomas [1]. Physical, chemical and biological carcinogens are thought to be responsible for lung cancer development, with tobacco smoking being the most prevalent (World Health Organization, 2021). However, epidemiologic data show that up to 25% of lung cancers can develop in nonsmokers [1,2]; in these patients, the development of cancer has been linked with specific driver genetic alterations, with the epidermal growth factor receptor (EGFR) gene being the most common globally [1,3,4].

Activating mutations of the *EGFR* gene have been reported pre-dominantly in patients who have not consumed tobacco or are considered light smokers, as well as in female patients and those of Asian origin. The *EGFR* gene, broadly expressed in normal tissues, was discovered in 1962 and has been found to be expressed in more than 60% of NSCLC cases as well as in squamous head and neck cancers and colorectal malignancies [3]. Later, *EGFR* was associated with the development of specific EGFR tyrosine kinase inhibitors (EGFR-TKIs), producing substantial responses and improving clinical outcomes in NSCLC patients whose tumors harbor activating mutations in the *EGFR* gene (namely, exon 19 deletions and exon 21 L858R point mutation) [4,5,6]. These activating *EGFR* mutations lead to alterations in ligand-dependent cellular signaling promoting cell survival, proliferation and continuous replication of cancer cells. EGFR TKIs act through competing with binding to adenosine triphosphate (ATP) of EGFR and inhibit phosphorylation, thus deactivating the gene and avoiding the initiation of the malignant transformation. All NSCLC-related EGFR mutations are clustered across exons 18–22 that encode the tyrosine kinase domain. [7,8]. While previous studies have reported the activity of first- and second-generation EGFR-TKIs, such as gefitinib erlotinib, afatinib and dacomitinib, as well as the third-generation inhibitors, osimertinib and furmonertinib, research efforts have focused on prolonging overall survival (OS) and improving quality of life in these patients with oncogene-addicted tumors [4,6,9,10,11,12,13].

Resistance to TKIs can be classified into two major categories; intrinsic and acquired. Intrinsic resistance accounts for 20–30% of cases and is related to poor initial response to TKIs [14]. It can be attributed to pre-existing de novo EGFR-dependent or independent mechanisms, whereas the acquired resistance can arise under therapeutic selective pressure, as a result of either the expansion of pre-existing subclonal populations or the evolution of drug-tolerant cells. That can be conceptualized through two distinct evolutionary pathways based on spatial and temporal heterogeneity. The former is defined as the uneven distribution of genetically diverse tumor subpopulations, whereas the latter represents the gradual adaptive response of cancer cells to TKIs. This heterogeneity of cancer cells may constitute the cornerstone of the observed resistance and seems to have a considerable contribution to treatment failure in NSCLC [15]. The combination of Darwinian selection and the innate diversity of cancer cells, as well as its clinical sequelae, appears as a hard-to-untie Gordian knot. The spatial and temporal diversity of cancer cells within a single patient set additional challenges to personalized precision medicine, calling for continuous cellular and molecular-level surveillance and adequate adjustment of the treatment plan.

This review focuses on the latest updates in the field of EGFR-mutant advanced NSCLC treatment, addressing the EGFR-dependent and independent mechanisms of resistance to EGFR-TKIs, including *MET* and *HER* gene amplifications, the role of mutations coding for the *RAS* and *PIK3CA* genes, as well as other less common genetic alterations. To liaise molecular oncology with clinical research and practice, we also discuss the detection of resistant clones through liquid biopsy and treatment options and novelties with a particular focus on challenges surrounding CNS metastasis. 

## 2. Materials and Methods

**Figure 1:**
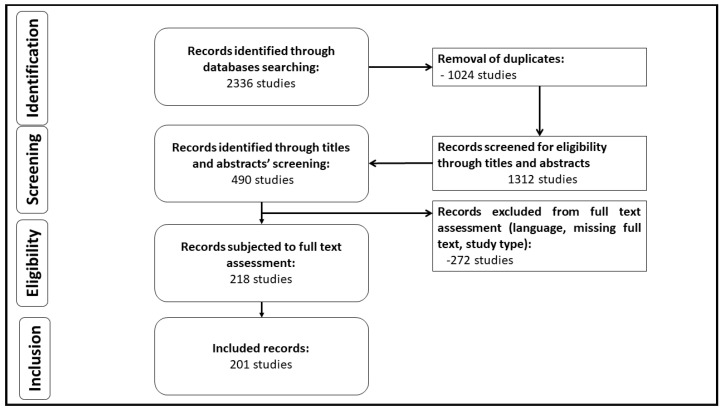
Literature search flow diagram.

Data collection was performed using PubMed, Ovid MEDLINE and Embase databases, with a defined search strategy from 2005 to date. Furthermore, the websites and online repositories of the World Health Organization (WHO) as well as the Cancer Research UK were used. All articles and pieces of news reported in scientific journals considering the advances in treatment with EGFR TKIs were considered. This research gave rise to approximately 2340 articles of which 201 references were deemed eligible for inclusion in this qualitative synthesis and discussion. Non-English language literature was excluded. Subject headings included (“Lung Cancer” or “NSCLC”) and (“EGFR-TKIs resistance” or Epidermal Growth Factor Receptor Tyrosine Kinase Inhibitors resistance), and/or “EGFR mutations”, and/or (“human epidermal growth factor receptor-2” or “HER2/erbB-2”), and/or “RAS mutation”, and/or “MET amplification”, and/or “Osimertinib”, and/or “T790M mutation” and/or “EGFR exon 20 insertions”, etc. Abstracts from the American Society of Clinical Oncology (ASCO), European Society of Medical Oncology (ESMO), and International Association of Lung Cancer websites were also reviewed. All reference lists for eligible studies were manually checked to ensure all relevant literature was retrieved. Two independent investigators performed the final selection based on the publication date, the impact factor of the journal, the relativity of the title and/or abstract, as well as the language of publication and duplicates were removed. The search end date was 11 June 2022 (Figure 1). 

## 3. Results

### 3.1. Mechanisms of Resistance

**Figure 2:**
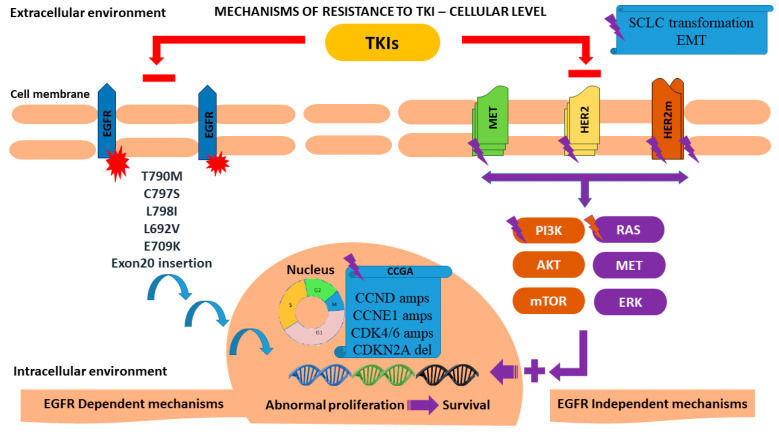
Mechanisms of resistance to EGFR-TKIs from a cellular perspective. Schematic representation of EGFR-dependent and independent mechanisms of resistance to TKIs. Stars and lightings illustrate protein mutations in the EGFR dependent and independent sides, respectively, red arrows represent inhibitory effects and purple and blue arrows depict activating effects. Resistance to TKIs decreases their inhibitory effect on intracellular signaling cascades associated with abnormal cell proliferation. EGFR: Epidermal Growth Factor Receptor, TKIs: Tyrosine Kinase Inhibitors, CCGA: Cell Cycle Gene Alterations, CCND amps: Cyclin D1 and Cyclin D2 genes amplifications, CCNE1: Cyclin E1 gene amplification, CDK4/6 amps: Cyclin-Dependent Kinase 4 and 6 genes amplification, CDKN2A: CDK inhibitor 2A, SCLC: Small Cell Lung Cancer, EMT: Epithelial Mesenchymal Transformation.

According to recent molecular studies, the concomitant occurrence of multiple driver mutations has been documented in a significant percentage of treatment-naive EGFR-mutant NSCLC. These findings seem to challenge the predominant concept of mutually exclusive driver mutations and explain why the majority of resistance mechanisms may be observed as either intrinsic or acquired patterns [14]. Various mechanisms inducing resistance to treatment with EGFR-TKIS have been reported, which can be further grouped into EGFR target-dependent and EGFR target-independent (Figure 2). 

### 3.2. EGFR Target-Dependent Mutations

#### 3.2.1. T790M Mutations

A secondary point mutation substituting methionine with threonine at amino acid position 790 (T790M) in exon 20 of the *EGFR* gene is a major contributor to TKI resistance, especially for first- and second-generation inhibitors. There is a twofold explanation for this acquired mechanism of resistance that can be found in 50–63% of post-TKI tumor samples. It has been shown that acquisition of the T790M mutation renders the ATP-binding pocket of the intracellular portion of the EGFR protein hostile to the reversible first-generation EGFR-TKIs, because the ATP affinity is restored to near wild-type levels in the L858R/T790M double mutant, thus resulting in failure of the drug to inhibit EGFR-mediated signaling. Additionally, the bulky methionine side chain causes steric hindrance, which affects the ability of first-generation inhibitors to bind to the ATP kinase pocket [16]. Two studies investigating tumor tissue or circulating DNA (ctDNA) of patients enrolled in the AURA3 trial associated this mutation in either blood or tissue samples with shorter progression-free survival (PFS) in patients who received first-line EGFR-TKI treatment [17,18]. 

Nevertheless, patients whose tumors maintained the T790M mutation after disease progression still had better outcomes compared to patients who lost the mutation according to a retrospective analysis of 143 NSCLC patients. Those who lost the T790M mutation after progression but maintained primary EGFR-activating mutations had a shorter median time to treatment discontinuation in comparison with those who maintained this mutation (6.1 months versus 15.2 months respectively; log rank *p* = 0.01) [19]. A study on 31 T790M positive NSCLC patients with progression after first-line EGFR TKIs yielded similar results; loss of T790M was associated with early progression and decreased survival [20]. Another retrospective trial with 49 patients who received osimertinib for T790M-mutated acquired resistance to prior EGFR-TKIs demonstrated enhanced clinical outcomes in those with maintained T790M mutation or with EGFR-dependent resistance mechanism. Specifically, the T790M-retain arm achieved a median PFS (mPFS) of 9.3 months in comparison with 7.8 months in the T790M-loss arm (*p* = 0.053). Participants with EGFR-dependent resistance mechanisms showed a significantly improved mPFS compared to patients with alternative pathway activation (13.5 months versus 8.2 months, respectively; *p* = 0.012) [21]. It appears that the loss of the T790M mutation coincides with the development of EGFR independent resistance mechanisms (including *KRAS* mutations, *MET* amplification, small-cell transformation and gene fusion), leading to resistance to second-line TKIs [19]. 

According to the AURA study, no evidence of acquired EGFR T790M mutation in plasma samples after progression on osimertinib was documented [22]. In the subsequent, phase III, FLAURA trial, patients who had progressed after receiving erlotinib or gefitinib were eligible for crossover to osimertinib in the case of emerging T790M mutation. The median OS was 38.6 months (95% confidence interval [CI], 34.5 to 41.8) in the osimertinib arm and 31.8 months (95% CI, 26.6 to 36.0) in the arm treated with first-generation TKIs (hazard ratio for death, 0.80; 95.05% CI, 0.64 to 1.00; *p* = 0.046). Eventually, 47% of patients who initially enrolled in the comparator arm received osimertinib as their second-line treatment. It is noteworthy that a 20% lower risk of death has been documented in favor of the osimertinib group, even in the presence of this crossover. Although osimertinib has been established as the preferred frontline TKI in EGFR mutant NSCLC, the aforementioned crossover seems to be beneficial to the comparator group in terms of OS (31.8 months) based on an indirect comparison with previous clinical trials of first- and second-generation TKIs that have revealed a median OS between 18 and 28 months [23]. 

#### 3.2.2. C797S Mutation

C797S is a tertiary mutation in *EGFR* in exon 20 where Serine takes the position of Cysteine at codon 797 of the ATP—binding site. From a mechanistic point of view, this decreases the ability of osimertinib and other TKIs (rociletinib, olmutinib, narzatinib) to form a covalent bond with the mutant EGFR [24,25]. It has been detected in up to 26% of patients with progression on second-line osimertinib [26] and to 7% of patients treated with osimertinib as a first-line treatment, representing the most frequent on-target mechanism of resistance to osimertinib [22].

A clinical study investigating the mutation profile of plasma samples of 93 advanced NSCLC patients progressing on osimertinib detected resistance-inducing mutations in 31 patients, 24% of whom carried the C797S mutation. In 2 patients, C797S coexisted with C797G mutations [25]. Nevertheless, other clinical studies assessing the mutational underpinnings of resistance to TKIs reported a consistently lower number of patients with C797S, thus challenging previous findings [27,28,29,30]. 

The rare co-occurrence of C797S with T790M mutations makes the use of first and third-generation TKIs alternately possible as a treatment strategy [31,32]. From a biological perspective, the identification of the in trans or in cis status of C797S and T790M mutations is of paramount importance because the subsequent therapeutic interventions depend on this status. Particularly, the combination of gefitinib and osimertinib seems to be effective in the case of synchronous exon19del/C797S and exon19del/T790M mutations on different EGFR alleles of the same cell (in trans). On the other hand, triple mutant (in cis) EGFRs with concomitant exon19del, T790M and C797S mutations are resistant to first-, second- and third-generation EGFR TKIs, as well as to their combinations [24]. The clonal evolution of C797S from in trans to in cis has also been considered as a potential mechanism of resistance to the combination of erlotinib and osimertinib [32]. Currently, after progression on third-generation TKIs, a re-biopsy might be recommended as a means to determine whether the C797S mutation is in cis or trans with T790M, as well as the subsequent management [24]. 

Recently, fourth-generation EGFR-TKIs have been developed with the aim of overcoming the acquired resistance caused by EGFR C797S tertiary mutation. LS-106 and BLU-945 are included in this category and have already demonstrated preclinical in vitro and in vivo antitumor efficacy in C797S–triple-mutant tumor models (EGFR19del/T790M/C797S and EGFRL858R/T790M/C797S) [33,34]. The phase I/II clinical trial, SYMPHONY, is currently recruiting EGFRm NSCLC patients who progressed after previous treatment with TKIs in order to estimate the Recommended Phase II Dose (RP2D) of BLU-945 as monotherapy based on its efficacy and safety results, as well as its RP2D when it is administered in combination with osimertinib [34]. Further research is needed to establish the clinical utility of fourth-generation EGFR-TKIs. 

#### 3.2.3. Rare EGFR-Dependent Mutations

Several studies have shed light on rare novel secondary resistance mutations on EGFR inhibition with TKIs. Rare EGFR mutations are often detected simultaneously as complex mutants, and their frequency is estimated at 1–8% [14]. Specifically, *EGFR* L718Q and L792X mutated residues have been detected by next-generation sequencing (NGS) in samples from 99 NSCLC patients experiencing clinical resistance to osimertinib [35]. The former accounts for 2% of osimertinib-resistant cases, whereas the latter up to 3% [22]. The same mutations were detected in NSCLC cfDNA samples which have been linked to resistance to osimertinib [25]. In vitro studies have confirmed the association between these mutations and resistance to osimertinib [36]. The co-existence of L792F/H mutations with G796S/R and C797S/G has also been connected to osimertinib resistance in NSCLC patients [35]. Additionally, a recent case series suggested that L792X and L718X mutations may also be resistant to dacomitinib—a second-generation TKI [37]. 

From a molecular standpoint, L792 mutations seem to drastically affect the binding of osimertinib on the hinge region of the kinase, between the COOH-terminal lobe and the smaller NH2-terminal lobe. This region is responsible for the formulation of hydrogen bonds to the adenine moiety of ATP. In silico experimentation, L792 hinge-pocket mutations, such as the most frequent L792H, have been reported to cause steric interference with a methoxy group on the phenyl ring of osimertinib [35]. The co-occurrence of these mutations with other EGFR-dependent alterations is a common phenomenon. A striking example is that they can be simultaneously observed in cis with T790M but in trans with G796/C797, regarding the same patient. Interestingly, gefitinib seems to retain its efficacy in L792 mutations according to in vitro studies. L718Q is another osimertinib-resistant mutation that seems to be sensitive to first and second-generation EGFR-TKIs in the absence of T790M mutation. L718Q residue is also located in the ATP-binding site inducing steric hindrance, thus affecting osimertinib-binding to the ATP-kinase pocket [25,38].

Other point mutations have been identified in small-series or single-patient case reports or have been identified by means of protein structure prediction models. These include a G796D mutation in a patient exhibiting resistance to second-line osimertinib [39], a G724S mutation affecting the P-loop of the EGFR kinase and G796R, G796S, G796D that may interfere in the binding of TKIs to EGFR conferring milder resistance. As far as the G724S-mediated resistance is concerned, it is characterized by an allele-specific pattern and is usually observed in ex19del but not L858R. Interestingly, afatinib seems to maintain its kinase affinity in G724 mutations [40,41]. Between G796R and G796S, the former has a more significant impact on the binding site. Nevertheless, relevant clinical evidence is missing [42]. 

#### 3.2.4. EGFR Exon 20 Insertions

Mutations and mechanisms that require further attention include exon 20 insertion and EGFR gene amplification, which if combined can greatly increase the abundance of mutated kinases accelerating the induction of resistance [17,43]. These de novo alterations have been considered responsible for primary or intrinsic resistance to the majority of EGFR-TKIs. They are highly enriched in Asian, nonsmokers, females’ adenocarcinomas, they are associated with poor outcomes, and their frequency has been estimated between 4 and 10% of all documented EGFR alterations in NSCLC [44]. They can be defined as small in-frame insertions or duplications of 3–21 base pairs that are clustered between 762 and 774 amino acid positions of EGFR protein, and they are considered to be largely mutually exclusive with other oncogenic driver mutations in NSCLC [45]. 

According to the oncogene addiction model, the inhibition of the EGFR signaling cascade can lead to rapid apoptosis in cells harboring classical EGFR alterations, such as exon 19 deletions and L858R mutations. These mutations induce conformational changes that destabilize the inactive form of the receptor towards an active state, which allows its ligand-independent dimerization and the stimulation of downstream signaling pathways, including the Ras/Raf/Mitogen-activated protein kinase (Ras/MAPK) pathway, phosphatidylinositol 3-kinase/AKT (PI3K/AKT and signal transducers and activators of transcription (STAT) pathways [46]. As it has already been mentioned, these classical mutations markedly reduce the ATP-binding affinity compared to the EGFR wild-type tumors in favor of ATP-competitive EGFR inhibitors [47]. On the other hand, these ATP-affinity modifications are not demonstrated in the case of exon 20 insertions, probably due to structural alterations of the C-helix. Particularly, they activate the receptor by pushing the C-helix from the C-terminal side, whereas exon 19 deletions contribute to the inward active conformation of the helix by pulling it from the opposite N-terminal side [48]. Consequently, the aforementioned insertions can confer de novo resistance to TKIs via both steric hindrance of TKIs binding and a constantly active EGFR conformation, irrespective of the presence of ligand binding, owing to the inward position of the regulatory C-helix, without modulating the ATP affinity for the benefit of TKIs [49]. 

**Figure 3:**
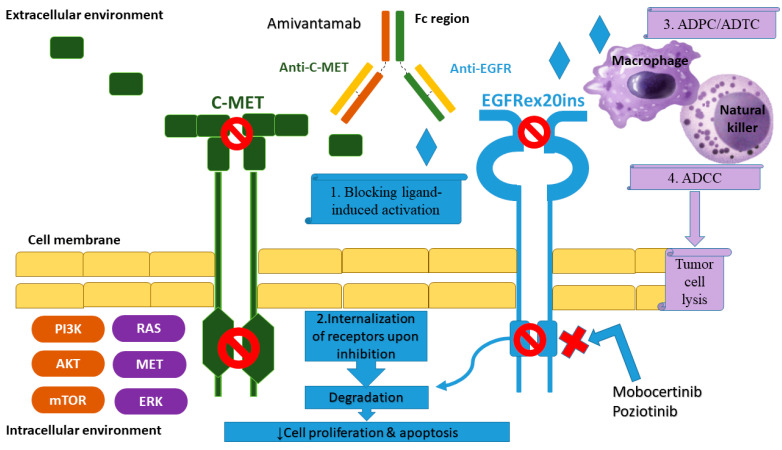
Mechanism of action of amivantamab in EGFR exon 20 insertions. Amivantamab is a human bispecific antibody, which is effective against EGFR with Exon 20 Insertion mutations. Additionally, amivantamab targets the C-MET membrane receptor, which mediates MET amplification, an emerging non-EGFR dependent mechanism of resistance. It yields a quadruple mechanism of action. First of all, it induces Fc independent downregulation of oncogenic signaling by means of downmodulation (1) and/or internalization of EGFR and C-MET membrane receptors and subsequent degradation that leads to apoptosis (2). Its immune-mediated activity is induced by macrophages-mediated ADCT or ADPC (3) as well as ADCC, which is primarily mediated by natural killers. Mobocertinib and poziotinib are additional novel agents with potential activity against NSCLC with EGFR exon 20 insertions. Mobocertinib is an irreversible TKI that selectively targets in-frame EGFRex20ins mutations and poziotinib is a pan-HER irreversible TKI. ADTC: Antibody-Drug Cellular Trogocytosis, ADPC: Antibody-Drug Cellular Phagocytosis, ADCC: Antibody-Drug Cytotoxicity.

Various experimental treatments are under development to overcome intrinsic resistance of exon 20 insertions to approved TKIs, including osimertinib. Whilst chemotherapy with or without immunotherapy remains the cornerstone of first-line treatment in this setting, there are early-phase clinical trials of novel agents that bear favorable results for the subsequent lines [50]. In particular, amivantamab is a bi-specific MET and EGFR antibody that overcomes the resistance of TKIs via binding to the extracellular domain of both receptors. Its immune-mediated activity is induced by multiple mechanisms, such as antibody-drug cytotoxicity (ADCC), which is primarily mediated by natural killers, macrophages-mediated antibody-drug cellular trogocytosis or phagocytosis (ADCT/ADPC) and the blockade of the ligand-induced activation that results in apoptosis, as well as the antibody (Fc)—independent downregulation of oncogenic signaling through heterodimerization, internalization and degradation of the EGFR-MET dimer (Figure 3) [51]. The activity of this agent was initially evaluated in the phase I CHRYSALIS (NCT02609776) trial. In this three-cohort trial, patients with metastatic postplatinum EGFRm NSCLC received either amivantamab monotherapy or amivantamab plus platinum-based chemotherapy or lazertinib, a third-generation TKI. The objective response rates (ORR) in the subgroup of patients with exon 20 insertions were 40%, the median duration of response (DoR) was 11.1 months, the median PFS was 8.3 months and the median OS was 23 months [52]. 

Another promising agent is mobocertinib, an irreversible TKI targeting EGFR and HER2 exon 20 variants, which has been tested in pretreated NSCLC patients with EGFR exon 20 insertion mutations [49]. A pooled analysis of phase I/II trials revealed ORR 28%, disease control rate (DCR) 78 and median PFS 7.3 months [53]. Moreover, it is worth mentioning that the presence of brain metastases reduces the ORR by approximately 50% (from 56% in patients without CNS involvement to 25% in patients with brain disease) [54]. Recently, savozertinib (DZD9008) has been granted breakthrouth therapy designation by FDA for patients with EGFR exon 20 insertions based on a phase I/II trial that showed ORR 48.4% (15/31 patients) and DCR 90.3% (28/31 patients) [55].

Additional experimental approaches are under investigation. A striking example is poziotinib, a pan-HER irreversible TKI, which showed ORR 43% and median PFS 5.5 months regarding the aforementioned subgroup of patients. Nevertheless, severe (grade 3 or higher) treatment-related adverse events (TRAEs), mostly diarrhea, rash and pneumonitis, were observed in 56% of patients [56]. This unfavorable safety profile may raise unsolved questions about its clinical utility. Significant toxicity has also been documented in combinations of the anti-EGFR monoclonal antibody cetuximab with EGFR-TKIs afatinib or osimertinib), leading to a potential negative clinical impact on these patients [57,58,59,60,61]. However, some of the aforementioned treatments, such as amivantamab, may modify the therapeutic strategy in the first-line setting, which is still similar to the management of NSCLC without driver mutations. Remarkably, the encouraging results of the Chrysalis trial led to the ongoing PAPILLON trial which compares the efficacy and safety of amivantamab in combination with chemotherapy in the first-line setting of NSCLC with EGFR exon 20 insertions [62,63].

### 3.3. EGFR Independent Pathways

**Figure 4:**
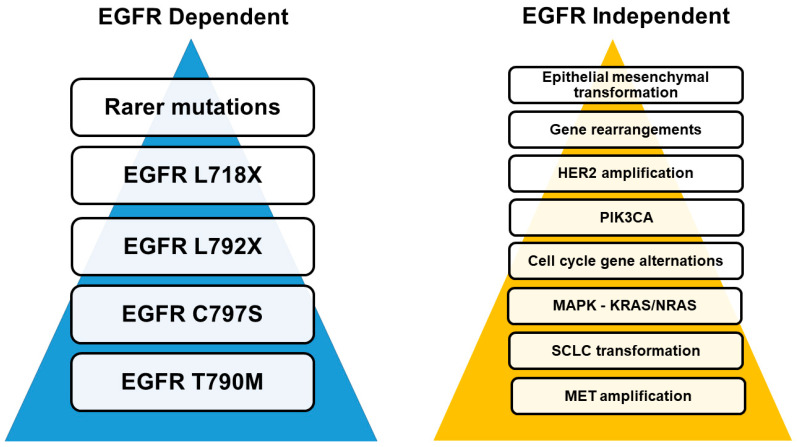
An overview of mutations involved in TKIs resistance. They are divided into EGFR-dependent mechanisms (on the blue pyramid) and EGFR-independent mutations (on the yellow pyramid). They are sorted in ascending order based on their frequency. The most frequent aberration lies at the bottom of each pyramid. SCLC: Small Cell Lung Cancer.

*EGFR* independent resistance mechanisms encompass a multitude of resistance pathways that either act exclusively or in combination with other EGFR dependent and independent pathways (Figure 2 and Figure 4). The majority of these mechanisms are not exclusive to NSCLC [64,65,66,67]. Below, the mechanistic background of these pathways is described. Table 1 provides an account of studies reporting EGFR independent resistance to TKIs.

#### 3.3.1. MET Amplification

*MET* is a proto-oncogene encoding a tyrosine kinase receptor, which is related to cell proliferation, survival, and migration, and it has been shown to play a multifactorial role in TKI resistance in NSCLC. Its amplification results in EGFR—independent activation of numerous signaling pathways, which are physiologically activated by EGFR in the context of NSCLC (mitogen-activated protein kinase—MAPK, signal transduction and activator of transcription—STAT, phosphatidylinositol 3-kinase—PI3K–Akt). Therefore, despite the pharmacological inhibition of EGFR, collateral pathways of carcinogenic activity do not cease to operate. *MET* amplification has been found to co-exist with T970M mutations promoting resistance in a synergistic manner [64,65,66,67]. 

MET amplification constitutes the most common resistance mechanism to osimertinib, providing a bypass pathway, and it should be distinguished from MET exon 14 skipping mutations. The former accounts for approximately 15% of acquired resistance to third-generation TKIs and is related to a worse prognosis, as well as requiring a different therapeutic strategy [68]. The latter is more frequent as a de novo driver mutation (about 3% of lung adenocarcinomas); however, it has been reported as a rare mechanism of acquired resistance to osimertinib in some case reports [69,70]. The combination of crizotinib and osimertinib has been evaluated in preclinical studies resulting in overwhelming resistance to osimertinib in cells with MET amplification; thus, it could constitute a potential treatment approach at the time of acquired resistance to third-generation TKIs in the near future [68]. The aforementioned bi-specific antibody against EGFR and MET, amivantamab, with or without lazertinib may also enrich the current therapeutic armamentarium against these molecular alterations [62]. On the contrary, as far as MET exon 14 skipping is concerned, the MET inhibitors, capmatinib and tepotinib, have already been approved in the first-line setting, and other multi-targeted TKIs, such as crizotinib and cabozantinib may also be considered as second- or later-line treatment choices [71,72,73]. 

Savolitinib, a selective MET inhibitor, has acquired increasing attention concerning MET alterations. Patients with MET-amplification, EGFRm NSCLC after progression on EGFR-TKIs are eligible for the ongoing phase II SAVANNAH trial of the combination of osimertinib plus savolitinib. This trial is based on the results of a previous Ib study, which has yielded an acceptable safety profile and a promising antitumor activity for this combination [74,75]. In particular, ORR was 30%, when these agents were administered to patients pretreated with third-generation EGFR-TKI, whereas a higher ORR was documented, roughly 65%, in third-generation TKIs-naive patients regardless of T790M mutational status. However, complete responses have yet to be observed in any group of patients [75]. Another phase II trial evaluated the activity and toxicity of monotherapy with savolitinib in participants with pulmonary sarcomatoid carcinomas and other NSCLC harboring MET exon 14 skipping alterations. An acceptable toxicity profile and a promising activity were demonstrated in terms of mPFS (6.8 months; 4.2 to 9.6), 1-year PFS (31.9%; 20.3 to 44.2) and independent review committee ORR (42.9%; 31.1 to 55.3) despite the absence of confirmed complete responses [76]. 

However, these agents have yet to be approved by the FDA for patients with MET amplification. Currently, capmatinib and crizotinib seem to be the most effective treatment options in patients with acquired resistance to TKIs who have progressed on chemotherapy with or without immunotherapy [72]. Particularly, capmatinib showed higher ORR in patients with MET high Gene Copy Number (GCN). In a phase I trial of patients who had received two or more prior lines of treatment, ORRs were 47% in those with GCN ≥ 6 compared to 25% in those with GCN 4–6 and only 6% in patients with less than 4 GCN. The mPFS was 9.3 months for the first group of participants [77]. Similar results were shown in a single-arm trial of crizotinib-treated patients. Observed ORRs were 38% in patients with high MET amplification (≥4 MET-to-CEP7 ratio) and the median PFS was 6.7 months [78]. 

#### 3.3.2. HER2 Amplification and Point Mutations

The human epidermal growth Factor Receptor 2 (*HER2*) gene encodes ErbB2, a TKI receptor of the EGFR family and regulates the downstream activation of several oncogenic pathways in the NSCLC context (extracellular signal-regulated kinase—ERK, MAPK, PI3K–Akt) [17,47]. Its effect can be enhanced when it coexists with other EGFR-dependent and -independent mutations. So far, its coexistence with EGFR L792X, EGFR C797X and/or PIK3CA amplification, EGFR G796S and MET amplification has been documented [68]. HER2 amplification and HER2 point mutations are responsible for 2–5% and 1.5% of acquired resistance to osimertinib, respectively. Exon 20 insertions, which are associated with the kinase domain, constitute the majority (up to 90%) of HER2 mutations [47].

A plethora of clinical trials are being conducted as a means to address the emerging therapeutic challenges of these molecular aberrations. Patritumab deruxtecan is a novel antibody-drug conjugate (ADC) that targets tyrosine-protein kinase ERBB3 receptor (HER3). This HER3 antibody is attached through a tetrapeptide-based cleavable linker to a toxic payload expressing the activity of topoisomerase I inhibitors. Its efficacy has initially been assessed in EGFR mutant patients failing upfront TKIs irrespective of the mechanism of TKI resistance. Specifically, the confirmed ORR was 39% [95% confidence interval (CI), 26.0–52.4], and the mPFS was 8.2 (95% CI, 4.4–8.3) months. Given that significant activity was demonstrated even in tumors with an agnostic mechanism of resistance to EGFR TKIs, this novel agent could potentially be integrated into the future algorithm of EGFR mutant NSCLC as a third-line treatment after progression of the disease on TKIs and platinum-based chemotherapy with or without bevacizumab and atezolizumab (IMpower 150) [79,80,81,82].

As far as HER2-amplified NSCLC is concerned, early clinical trials have not yielded any significant benefit. However, according to a separate interim analysis of the DESTINY-Lung01, fam-trastuzumab deruxtecan has demonstrated a 24.5% (95% CI, 13.3–38.9%) ORR in HER2-overexpressed, immunohistochemistry (IHC) 2+ or 3+, HER2 mutation-negative NSCLC, which may be related to HER2 amplification. mPFS was 5.4 months (95% CI, 2.8–7.0 months). The initial design of this 2-cohort phase 2 trial included a patients’ group with HER2-overexpressing (IHC 3+ or IHC 2+) and another group with HER2 mutations [83]. Trastuzumab deruxtecan is another ADC that has initially been evaluated in other malignancies, such as breast and gastric cancers. It is composed of a humanized anti-HER2 antibody, a cleavable linker and a cytotoxic topoisomerase I inhibitor [84,85,86].

The molecular association between HER2 amplification and HER2 mutations has yet to be distinctly defined [87]. Despite the limited choices in the case of HER2 amplification, genetic engineering has ushered in a new epoch of abundant novel targeted options regarding HER2 mutations. The implementation of these emerging off-label approaches could be beneficial after progression on chemotherapy-based regimens; therefore, patients with these mutations should be encouraged to participate in clinical trials of anti-HER2 agents. Phase II trials of ADCs, such as ado-trastuzumab emtansine (T-DM1), which incorporates the anti-HER2 actions of trastuzumab with the microtubule inhibitor DM1 and fam-trastuzumab deruxtecan, have shown favorable outcomes in terms of ORR. The former revealed a 44% (95% CI, 22% to 69%) ORR and its median PFS was 5 months (95% CI, 3 to 9 months), as stated by a phase II basket trial, whereas the latter showed confirmed ORRs 55% (95% confidence interval [CI], 44 to 65), median PFS 8.2 months (95% CI, 6.0 to 11.9) and median OS 17.8 months (95% CI, 13.8 to 22.1) [85,88]. Toxicity was generally consistent with previous clinical data. The most common TRAE was neutropenia (19%), and 46% of patients experienced grade 3 or higher TRAE, including grade 5 pneumonitis (2%). It is noteworthy that efficacy results were consistently documented across subgroups, including participants with CNS involvement, and the reported anticancer activity was demonstrated across different HER2 mutation subtypes, as well as in patients with HER2 amplification or undetectable HER2 expression [85].

Plenty of additional targeted treatments have yielded promising results concerning HER2-mutated NSCLC. Pan-HER inhibitors, such as afatinib, neratinib, dacomitinib, tarloxitinib and pyrotinib, anti-HER2 agents, such as trastuzumab, lapatinib and pertuzumab (which also inhibits ligand-dependent HER2–HER3 dimerization), poziotinib, which is a covalent, irreversible and potent inhibitor of EGFR and HER2 exon 20 insertions, as well as the aforementioned mobocertinib combined with T-DM1 are some agents that have been evaluated in this setting [47]. In the European EUHER2 cohort, the overall RR of patients who received anti-HER2 targeted treatment, including trastuzumab, neratinib, afatinib and lapatinib was 51%. The arm which was treated with the combination of trastuzumab and chemotherapy compared to those treated with afatinib demonstrated RRs 50% versus 18% and PFS 5.1 months versus 3.9 months, respectively [89,90]. The recently published IFCT-1703 R2D2 trial evaluated the combination of trastuzumab, pertuzumab, and docetaxel in pretreated NSCLC patients with HER2 exon 20 insertions and point mutations. It is worth mentioning that 30% of them had initially been presented with brain metastases. The ORR was 29% and the median PFS was 6.8 months (95% CI, 4.0 to 8.5) [91]. Poziotinib gained FDA fast track designation in pretreated NSCLC patients with HER2 exon 20 insertion mutations in March 2021 based on the results of the ZENITH20 trial. The confirmed ORR was 27.8% (95% CI, 18.9 to 38.2), the DCR was 70% (95% CI, 59.4 to 79.2), the median DoR was 5.1 months (95% CI, 4.2 to 5.5) and the median PFS was 5.5 months (95% CI, 3.9 to 5.8) [47,92]. Pyrotinib has been evaluated in three phase II trials of pretreated HER2 exon 20-mutated advanced NSCLC. ORRs were ranged from 30% to 53.3%, the maximum estimated mPFS was up to 6.9 months, and the estimated mOS was up to 14.4 months (12.3–21.3) [93,94]. Finally, Tarloxotinib is a pro-drug that releases its active metabolite under hypoxic conditions. The active tarloxotinib-E is a potent irreversible pan-HER TKI that demonstrated 22% ORRs and 66% DCRs in HER2-mutant NSCLC [47,95]. In conclusion, anti-HER2 antibodies, chemotherapy and immunotherapy seem to be limited-benefit therapeutic approaches for NSCLC patients with HER2 alterations, whereas ADCs (Trastuzumab deruxtecan and T-DM1) and TKIs, such as poziotinib and pyrotinib, are emerging treatment options [96]. 

### 3.4. MAPK–KRAS/NRAS

The RAS–MAPK pathway physiologically mediates cellular signaling between the extracellular milieu and the nucleus, activating genetic interactions related to cell growth, division and differentiation. A diverse set of mutations and aberrations can lead to RAS–MAPK mediated oncogenesis or resistance. Preclinical studies on NSCLC cells resistant to TKIs have reported two types of NRAS mutations—namely E63K, a novel single base pair substation, and a gain of copy number of wild type (wt) NRAS or wt KRAS [97]. Combinations of osimertinib and BRAF inhibitors, such as encorafenib, are investigated in this molecular setting, whereas favorable outcomes have recently been demonstrated regarding the combination of dabrafenib, another BRAF inhibitor and trametinib, a MEK inhibitor even in treatment-naïve BRAF-mutant patients [68,98]. Specifically, this double inhibition was associated with an ORR of 68% in pretreated patients and 64% in the first-line setting [98].

#### 3.4.1. PIK3Cam

PIK3CA mutation or amplification and deletion of the tumor-suppressing phosphatase and TENsin homolog deleted on chromosome 10 (PTENS) gene can activate signaling cascades downstream to the PI3K pathway [43]. These events can co-occur with other resistance-associated mutations in NSCLC leading to double mutant NSCLC with 20–30% shorter progression interval and 20% lower response to treatment as shown by Eng and colleagues [99]. There are ongoing clinical trials that evaluate the efficacy of the PI3K inhibitor alpelisib [100] and the combination of EGFR TKIs with the mTOR inhibitor everolimus [101]. 

#### 3.4.2. Gene Rearrangements

Gene rearrangements lead to the formation of hybrid genes possessing parts of the combined genes and different functions. The oncogenic potential of fusions was initially identified in rare types of leukemia and sarcoma [102]. To date, they have been detected in the pathogenesis of more common types of cancer including NSCLC, where gene fusions account for 3–10% of cases of acquired resistance to TKIs [103]. The most common NSCLC fusions that are considered responsible for acquired resistance to third-generation TKIs include SPTBN1-ALK, RET–ERC1 and FGFR3–TACC3, with CCDC6–RET, NCOA4–RET, NTRK1–TPM3, AGK–BRAF, GOPC-ROS1 and ESYT2–BRAF. It appears that fusions may co-occur with EGFR dependent (EGFR C797S) and independent (BRAF mutation and MET amplification) mechanisms of resistance [18,69,104].

#### 3.4.3. Genetic Aberrations in the Cell-Cycle Related Genes

Cell-cycle gene alterations are supposed to be responsible for approximately 10% of the acquired resistance to osimertinib and have been associated with unfavorable outcomes [104]. These alterations include mainly amplifications in the genes that encode cyclin D1, D2, E1, cyclin-dependent kinase (CDK) 4 and 6, as well as deletion frameshift mutations in the gene of the CDK inhibitor 2A [68]. Both preclinical and clinical studies have already been designed to assess the efficacy of CDK4/6 inhibitors, such as abemaciclib, with or without osimertinib (ClinicalTrials.gov, NCT04545710). The rationale of this approach is that CDK4/6 kinases are responsible for the phosphorylation of the retinoblastoma protein (Rb), which plays a crucial role in multiple genetic events that sustain cell proliferation leading to the resistance to TKIs. Therefore, the phosphorylated Rb could be a potential predictive biomarker, as well as a novel target in case of emerging resistance to osimertinib. Recent preclinical data support that Rb phosphorylation is maintained in the majority of NSCLC cell lines with either intrinsic or acquired resistance to osimertinib, and thereby, CDK4/6 inhibitors represent an emerging therapeutic option [105]. According to preclinical studies, the combination of osimertinib with abemaciclib or palbociclib has been shown to downregulate the Rb phosphorylation, block the resistant cells in the G1 phase and harness the cell proliferation [106]. Ongoing clinical trials will address whether the combination of CDK4/6 inhibitors and osimertinib can thwart the resistance to third-generation TKIs.

#### 3.4.4. NSCLC to SCLC Transformation

It has been estimated that 4–15% of resistance to first-line TKIs cases are associated with histologic transformation from NSCLC to SCLC. WGS of histological samples from NSCLC tumors that underwent SCLC transformation under EGFR-TKI treatment revealed complete inactivation of tumor-suppressing genes (*RB1*, *TP53*) in both the initial NSCLC and at the newly detected SCLC. Combined with the aforementioned, the presence of apolipoprotein B mRNA editing enzyme was also linked to the histological transformation [107]. A plethora of case reports and studies from various international clinical centers have confirmed the existence of the mechanism and reported its detrimental clinical outcomes [108,109,110,111,112,113]. 

#### 3.4.5. Epithelial-Mesenchymal Transition (EMT)

Resistant cells expressing EMT features without secondary EGFR mutations have been identified in patients who progressed on gefitinib or osimertinib. In particular, the observed EMT features include the decrease in the major component of the adherens junctions, E-cadherin and the overexpression of the mesenchymal biomarker, vimentin, as well as of the E-cadherin degradation-inducer, Hakai [114,115]. During this process, epithelial cells lose their polarity and adhesion and their acquired mesenchymal phenotype potentiates cellular migration. Zeb, Snail, Slug, and Twist are included in the transcriptional factors that regulate the E-cadherin expression, and they are highly considered potential therapeutic targets [116].

As far as Snail is concerned, it facilitates the transformation of malignant epithelial cells into stromal cells and the cell migration by enhancing their invasion ability. Preclinical data support that palbociclib can overcome the resistance to EGFR-TKIs in EGFR-mutant NSCLC cell lines via genetic silencing of Snail or the downregulation of its expression [116]. The underlying mechanism might be attributed to the inhibition of the CDK4/6-mediated activation of the DUB3 deubiquitinase. DUB3 promotes Snail degradation and stabilization, as well as the cyclin A protein stabilization through the removal of the polyubiquitin chains from cyclin A, thus inducing cell-cycle progression for the proliferation of NSCLC [117]. Gefitinib sensitivity has also been demonstrated to be restored in resistant EGFR-mutant cell lines via Hakai knockdown, which leads to increased E-cadherin expression and attenuation of stemness. JMF3086 is a dual 3-hydroxy-3-methylglutaryl coenzyme A reductase (HMGR) and histone deacetylase (HDAC) inhibitor that is currently being investigated. Its mechanism of action is based on the Src/Hakai inactivation and the decreased interaction between Hakai and E-cadherin, which subsequently reverses the EMT-like features of the cells and their resistance to TKIs [114]. 

Furthermore, the overexpression of the EMT transcription factor TWIST-1 in NSCLC cells harboring EGFR mutations has been linked to acquired resistance to EGFR-TKIs. TWIST1-mediated resistance has been partially attributed to its direct binding to the intronic regions and promoter of the pro-apoptotic BH3-only gene, BCL2L11 (BIM), resulting in the suppression of its transcription. Consequently, given that TWIST1 seems to act as a driver of EMT-mediated TKIs resistance, TWIST1 and BCL2 inhibitors are currently being investigated as means to over-ride this resistance mechanism. Interestingly, harmala alkaloid, harmine, is a first-in-class TWIST1 inhibitor that contributes to growth inhibition and apoptosis in EGFR-mutant NSCLC cells [68,118]. An overview of mutations involved in TKIs resistance is presented in Figure 2 and Figure 4. 

**Table 1:** Studies reporting EGFR independent NSCLC resistance to TKIs mechanisms. PD: Progression of Disease, NSCLC: Non-Small Cell Lung Cancer, TKIs: Tyrosine Kinase Inhibitors, ctDNA: circulating tumor DNA, NGS: Next Generation Sequencing.

Resistance Mechanism(s)	Study Design	Outcomes	Reference
Amplification of MET, HER2, and PIK3CA	Analysis of plasma samples of 83 patients with PD on first-line osimertinib	MET: 14 samples—19%, HER2: 4 samples—5%, PIK3CA: 3 samples—4%	Papadimitrakopoulou et al., 2018 [103]
Mutations in AKT1, BRAF, ERBB2, KRAS, MEK1, NRAS and PIK3CA, MET and HER2	Molecular analysis of tumor samples from 155 patients with lung adenocarcinomas and acquired resistance to erlotinib or gefitinib	MET amplification in 4 samples, HER2 amplification in 3 samples	Yu et al., 2013 [119]
MET, EGFR, PIK3CA, ERRB2, KRAS, RB1	CAPP-Seq ctDNA analysis of 115 plasma samples from 43 patients to identify resistance-inducing mutations in 43 NSCLC patients treated with rociletinib	An increased copy number in MET or ERBB2 was detected in 14 patients (34%) in combination to EGFR mutations, single nucleotide variants (SNVs) in EGFR, PIK3CA or RB1 in 3 patients (7%) and an increased copy number in MET in combination with SNVs in PIK3CA or RB1 in 2 patients (5%)	Chabon et al., 2016 [120]
EGFR dependent and independent mutations	Amplicon-seq analysis on tissue samples of 20 NSCLC patients at PD or baseline treated with TKIs	MET amplification in 1 patient with brain metastasis after prolonged treatment with osimertinib	Martinez-Marti et al., 2017 [64]
EGFR dependent and independent mutations	Tumor biopsy analysis of 7 patients treated with TKIs (AZD9291 or rociletinib)	Recurrent MET or ERBB2 amplification in 5 patients with resistance to third-generation TKIs, KRASG12S mutation in one tumor resistant to AZD9291	Ortiz-Cuaran et al., 2016 [66]
EGFR dependent and independent mutations	Molecular profiling analysis at the time of PD in blood and tissue samples of 118 patients treated with TKIs	MET amplification in 14% of the patients, recurrent alterations detected in PIK3CA, EGFR, and RET of >3.3% of patients	Le et al., 2018 [121]
EGFR dependent and independent mutations	NGS on tumor tissue or blood samples of 117 patients with stage IIIb-IV EGFR-T790M NSCLC	MET amplification in 3 (33.33%) patients, BCL2L11 loss (BIM deletion polymorphism) in 1 (11.11%) patient, ERBB2 amplification in 1 (11.11%) patient, PTEN mutation in 1 (11.11%) patient, EZH2 mutation in 1 (11.11%) patient	T.S.K. Mok et al., 2019 [26]
EGFR dependent and independent mutations	NGS plasma samples’ analysis from 559 patients with previously untreated EGFRm advanced NSCLC treated with TKIs; osimertinib (*n* = 279), gefitinib or erlotinib (*n* = 277)	MET amplification in 14 patients treated with osimertinib and in 5 patients treated with gefitinib or erlotinib, HER2 amplification, PIK3CA and RAS mutations in 6 patients treated with osimertinib and 3 patients treated with gefitinib or erlotinib	Ramalingam et al., 2018 [22]
EGFR dependent and independent mutations	Molecular analysis of tumor tissue and plasma samples from 12 EGFR-mutant NSCLC patients before and after osimertinib treatment	KRAS G12D mutation in 1 patient, PIK3CA E545K mutations in 2 patients, pre-existing KRAS G12D mutation and PTEN loss in 2 patients with primary resistance to osimertinib	Hong et al., 2018 [122]

The majority of the available studies have used different samples (blood, tissue), taken at different time points (before, during, after the development of resistance or combination of the above) and different molecular analysis methods, which may have led to heterogeneous results. Several studies involved small numbers of patients (*n* < 10), making the detection of rare mutations more difficult, while other studies described rare or novel mutations based on case reports [123,124]. Rare EGFR independent mutations were in some instances reported [25,125]. Several studies reported an overlap of the prevalent EGFR-dependent mechanisms with EGFR-independent mechanisms—in these cases, evaluating the separate contribution of each of the mutations was not possible [120,121].

### 3.5. CNS Disease

Overall, understanding the molecular underpinnings of TKIs resistance in advanced NSCLC is essential in order to devise targeted therapeutic approaches. While examining the therapeutic challenges and options for each type of NSCLC metastasis exceeds the scope of the present review, the authors focus on NSCLC-related CNS disease. This type of metastasis affects up to 70% of patients with NSCLC harboring an oncogenic driver mutation during the course of the disease and is particularly resistant to treatment due to the anatomical intricacies of cerebral circulation [126]. Discussing therapeutic developments in this field encompasses a significant proportion of clinical studies related to the precise and personalized management of advanced NSCLC. 

A wealth of evidence suggests that CNS metastases require special attention in the context of EGFR mutant NSCLC. It has been estimated that up to 30% of patients with this type of lung cancer develop brain metastases (BM) during the course of the disease [127]. The incidence of BMs associated with EGFR mutant NSCLC considerably surpasses the incidence of BMs in NSCLC with driver aberrations, such as Anaplastic Lymphoma Kinase (ALK) rearrangements or in wt disease [128,129,130]. Specifically, the incidence of BMs in EGFR mutant tumors can reach up to 70% during the whole course of the disease, in contrast to 38% observed among patients with EGFR—wt NSCLC [131].

NSCLC BMs are more frequently detected among Asians (39–63%) in comparison to Europeans and North Americans (2–40%) [132,133,134,135,136]. Significant discordance between EGFR mutant NSCLC primary tumors and BMs has been detected in up to 22.4% of cases in small sample studies [135]. In this frame, the frequency of T790M mutation in BMs (17%) is lower than in primary tumors [137]. In a limited number of cases (4.1%) symptoms leading to the initial diagnosis stem from BMs. From a mechanistic point of view, it seems that NSCLC BMs development leads to structural changes of the adjacent BBB, leading to the formation of what has been coined as brain–tumor barrier (BTB). BTB tissue is characterized by dilated capillaries overexpressing CD31 and loss of both collagen IV and aquaporin-4 when compared to healthy BBB [138]. From a clinical standpoint, these metastases tend to be smaller in size but more disseminated in comparison to BMs associated with EGFR wt NSCLC [134], and they lead to worse outcomes in comparison to extracranial EGFR mutant NSCLC metastases [139].

From a cancer management perspective, chemotherapy was shown inferior to TKIs in a metanalysis of 11 clinical trials [140]. Brain radiation therapy may also be used [141,142], which seems equally effective among patients with TKIs resistant and non-TKIs resistant BMs [142]. Nevertheless, it should be emphasized that these patients tend to present at a younger age, compared to EGFR wild-type NSCLC. Given the excellent intracranial activity of newer-generation EGFR TKIs, the need for brain radiotherapy is increasingly obviated in asymptomatic patients [143]. The majority of EGFR-TKIs have been used and investigated for the treatment of EGFR mutant NSCLC BMs in preclinical and clinical studies (Table 2). 

Based on the available evidence, osimertinib has become the standard of care in EGFR-mutant advanced NSCLC with brain metastases to date. Dose escalation has been associated with more favorable outcomes, particularly in combination with radiotherapy without adding toxicity [151]. Remarkably, the BLOOM study evaluated osimertinib 160 mg once daily in EGFRm patients with cytologically confirmed leptomeningeal disease (LMD). Median PFS and mOS were 8.6 months (95% CI, 5.4–13.7 months) and 11.0 months (95% CI, 8.0–18.0 months), respectively. LMD ORR was 62% (95% CI, 45–78%), whereas overall ORR was 41% (95% CI, 26–58%). Cerebrospinal fluid complete responses were confirmed in 28% (95% CI, 15–44%) of patients, and neurologic function was enhanced in 57% of those with neurologic manifestations at baseline [149]. Encouraging evidence about EGFR-TKIs needs to be assessed in larger clinical trials. Simultaneously, a growing body of knowledge has emphasized the need to diversify the available treatment options by incorporating immunotherapy into later lines of treatment. 

#### 3.5.1. Immunotherapy and EGFR TKIs Resistance

Immunotherapy has also been explored in the post-EGFR TKI setting. Immune checkpoint inhibitors (ICIs), namely monoclonal antibodies against PD-1 and programmed death ligand-1 (PD-L1), have been used as post-TKI salvage treatment, alone or in combination with chemotherapy. Preclinical studies assessing the impact of anti-PD-(L)1 ICIs on cellular and animal models of EGFR mutant NSCLC have shown promising evidence [153,154]. Nevertheless, clinical trials and studies to date have not confirmed preclinical evidence, with patients harboring EGFR mutations having worse outcomes than patients with EGFR wt NSCLC on anti-PD-(L)1 ICIs [155,156,157]. 

These poor outcomes have been attributed to the lower levels of PD-1 molecule expression on EGFR mutant NSCLC cancer cells and to the complex interactions of EGFR mutant cells with the tumor microenvironment (TME). Ji and colleagues (2016) investigated the genomic profile of 100 resected NSCLC specimens and attempted to establish correlations between mutation status and PD/PD-L1 expression. EGFR mutations were correlated with lower PD/PD-L1 expression [158]. An in vitro study by Chen et al. (2015) showed that EGFR TKIs induced PD-PD-L1 expression in nonresistant NSCLC, but could not have a synergistic tumor cell killing effect with PD ICIs in EGFR mutant NSCLC with resistance to TKIs [154].

Regarding the tumor micro-environment impact on the therapeutic effectiveness, several studies have shown that EGFR mutations can increase the proliferation of T-regulatory cells and myeloid-derived suppressor cells (MDSCs) [159], whereas they can also downregulate tumor-infiltrating lymphocytes (TILs) [160,161], tumor-associated macrophages (TAMs), immunoregulatory cytokines [162] and exosomes [163]. Although this interaction is not directly related to the expression of PD/PD-L1 molecules, combined with the lower expression of PD/PD-L1 molecules, it can increase the immune escape potential of malignant cells.

The potential of PD/PD-L1 expression to provide prognostic or predictive insights into EGFR mutant NSCLC has also been investigated. Yang et al. (2020) reported that lower pre-treatment PD-L1 expression is associated with better ORR, PFS, in EGFR mutant NSCLC. However, the same was correlated with a higher rate of T790M resistance to EGFR TKI-treated lung AC [164]. A retrospective analysis of 600 tissue samples by Fan and colleagues (2019) indicated that the expression of PD/PD-L1 molecules can be higher in rare EGFR mutations, such as G719A, than in common EGFR mutations. The same study suggested that the presence of PD/PD-L1 molecules did not affect the behavior of these cells towards EGFR TKIs [165].

On the other hand, Chang et al. (2021) reported that PD-L1 expression was not associated with better or worse outcomes in a sample of 114 patients [166]. A recent meta-analysis on the matter, including 991 patients from 11 eligible studies, concluded that high PD-L1 expression is associated with shorter PFS. No association between PD-L1 expression and OS was shown, and there was no definite answer to the question whether PD/PD-L1 status can be used as a prognostic or predictive biomarker [167].

Additional concerns about the effectiveness of EGFR TKIs in NSCLC stem from their pharmacological composition. Low solubility, inconstant oral bioavailability and high binding potential to plasma albumin necessitate large daily doses and might contribute to the development of early resistance to EGFR-TKIs. Nanocarriers including liposomes, polymer nanoparticles, micelles, and nanogold particles have major potential to reverse these disadvantages [168]. The main advantages of nanocarriers are non-immunogenicity, biocompatibility, high drug loading capacity and controllable release of the loaded regimens. To date, preclinical studies have indicated significant lymph node uptake of hyaluronic acid-modified liposomes loaded with docetaxel and gefitinib in lung cancer models [169]. Similarly, liposomes loaded with erlotinib and quercetin, a natural compound, exhibited inhibitory activity over phosphorylation at both upstream and downstream of EGFR in in vitro NSCLC models. The latter indicates the potential of nanocarriers to provide multipotent combining agents increasing the therapeutic potential of the therapeutic regimen [170]. 

Given that up to 35% of NSCLC patients harbor intrinsic mutations which significantly impact response rates on first-generation TKIs, the development of nanodrugs that are designed to knock down both mutated genes and wild-type genes could be beneficial to overcome both intrinsic and acquired compensatory mechanisms of resistance [171]. A large number of bioactive inorganic nanoparticles (NPs), such as gold (Au) NPs, silver (Ag) NPs, metallic oxideare, as well as nonmetallic NPs, such as selenium NPs, are currently being investigated. A plethora of biomedical applications have been successfully demonstrated in vitro based on their exceptional properties, such as easy accumulation in cancer cells, fluorescence imaging, enhanced Raman scattering, photothermal and antimicrobial properties [172]. However, the capacity of nanoformulations to yield these results in clinical context has yet to be proved.

#### 3.5.2. Clinical Trials of Immunotherapy in EGFR-Mutant NSCLC

A recent phase II study invigorated the PD-L1 inhibition concerning the second-line treatment of metastatic EGFR mutant ACs after TKI failure. Specifically, the combination of carboplatin, pemetrexed bevacizumab and atezolizumab, which is an anti-PD-L1 agent, achieved promising efficacy with acceptable toxicity including patients with EGFR-mutant tumors irrespective of PD-L1 status. Specifically, in this group of patients, ORR was estimated at 62.5%, median PFS was 9.4 months (95% CI: 7.6–12.1) and one-year survival was 72.5% (95% CI: 0.56–0.83) [173].

The IMpower150 study also provides illuminating information concerning the efficacy of immunotherapy in EGFR-mutant patients. This trial was positive in terms of PFS and OS regarding the total population. Remarkably, 10% (124/1202) of participants were EGFR-positive and they were randomized into three arms. Thirty-four patients (9%) received the quadruple atezolizumab, bevacizumab (an anti-VEGF agent) plus carboplatin and paclitaxel (ABCP), 45 patients (11%) were treated with atezolizumab plus chemotherapy (ACP) and 45 patients (11%) enrolled in the standard bevacizumab plus chemotherapy group (BCP) [81,174]. A key subgroup analysis of patients harboring EGFR mutations showed that the OS Hazard Ratio for the ABCP arm versus the BCP arm was 0·61 (95% CI 0·29–1·28). mOS was not estimable (95% CI 17·0–NE) for the ABCP group versus 18.7 months (95% CI 13·4–NE) for those treated with BCP. However, the results were not statistically significant, probably due to the insufficient sample size and the subsequent reduced statistical power. It is worthy of note that only the quadruple ABCP is superior to BCP (HR; 0.61). ACP versus BCP failed to yield a similar benefit in EGFR-mutated patients [82]. These results may reflect the beneficial synergistic effect of anti-angiogenesis and immunotherapy. Another similar quadruplet that bears promising early results is currently being investigated in the phase III ORIENT-31 trial. Sintilimab, a novel anti-PD-1 agent with or without IBI305, a biosimilar of bevacizumab, plus chemotherapy in EGFRm NSCLC after progression on EGFR-TKI significantly prolonged mPFS (6.9 versus 4.3 months; HR 0.464, 95% CI: 0.337, 0.639; *p* < 0.0001) and increased ORR (43.9 versus 25.2%) in comparison with chemotherapy alone [175].

Furthermore, rechallenging EGFR TKIs after chemotherapy either with or without immunotherapy remains an unaddressed issue owing to the current lack of evidence. Successful responses to osimertinib rechallenge following intervening chemotherapy in EGFR T790M mutated adenocarcinomas have only sporadically been observed. In summary, further investigation regarding the optimal treatment algorithm after TKIs failure is needed [176]. 

Overall, immunotherapy in EGFR TKIs resistant NSCLC has had a limited clinical utility to date. Novel agents, such as REGN7075, in combination with immunotherapy are currently under evaluation. REGN7075 is a T-cell bi-specific antibody that binds to EGFR and engages T-cells via CD28 resulting in target cell killing by T-cell activation. Its combination with an anti-PD-1 ICI, namely cemiplimab, is currently being assessed by a phase I/II clinical trial l (NCT04626635) [177]. Further clinical trials are required to establish its potential immunostimulating and antineoplastic efficacy. As far as the clinician’s therapeutic strategy is concerned, ICIs combined with chemotherapy may consist of an alternative therapeutic approach, whereas it remains controversial whether rechallenging EGFR TKIs following a combination of chemotherapy and immunotherapy could be beneficial to some patients. Therapeutic prospects might improve as long as an early and precise diagnosis of EGFR TKIs resistance is achieved [178].

#### 3.5.3. Detection of EGFR TKIs Resistance by Means of Liquid Biopsy

Liquid biopsies are an emerging tool for the monitoring of solid malignancies, such as colon carcinoma, breast cancer, melanoma, and lung cancer including NSCLC [179]. The two most developed types of liquid biopsies are the circulating tumor cells (CTCs) and the circulating tumor DNA (ctDNA) [180]. Liquid biopsies can be used to monitor existing mutations or detect novel ones and provide insights into the prognosis and the progression of the disease, as well as the effectiveness of treatment [181]. They can be obtained through peripheral blood sampling with more safety, fewer complications and at a lower cost than tissue biopsies [182,183]. 

With respect to EGFR mutant NSCLC, liquid biopsies have been used to describe the genomic profile of tumors and/or to correlate the emergence of resistant clones with clinical progression on treatment with TKIs. Jori and colleagues (2021) have recently analyzed real-world data from 56 patients at the time of disease progression after the failure of osimertinib. Second-line osimertinib had been administered to 47 of those patients. Six plasma samples did not reveal the initial driver alteration. Thirty-seven of the remaining 41 participants (90%) had developed *T790M EGFR* mutations after the prior administration of first or second-generation TKI, while 39% developed EGFR C797S and 12% developed non-C797S EGFR mutations, such as L718Q, V843I, L792H, and C724S. They also reported mutations in multiple pathways, copy number changes and rare fusions of *RET*, *ALK*, *FGFR3* and *BRAF* leading to single and multiple TKI resistance mechanisms in single or smaller numbers of patients [184]. 

Fuchs and colleagues (2021) subjected liquid biopsies obtained from 30 patients to NGS and detected differences between the mechanisms of resistance to first and second-line TKIs. In particular, *MET* amplification was more common in resistance to first-line therapy, and C797S was more common in second-line treatment [185]. While the capacity of liquid biopsies to detect genetic alterations is explored, concerns have been raised about their sensitivity. According to a relevant meta-analysis, liquid biopsies obtained from the peripheral blood of EGFR mutant NSCLC patients have very high specificity (up to 98%), but lower sensitivity (around 68%) for the detection of mutations [186]. For this reason, it is feasible to monitor known mutations or detect novel ones with liquid biopsies. However, negative liquid biopsy results should be validated with tissue biopsies.

Liquid biopsies may also shed new light on the mechanisms of both primary and acquired resistance to EGFR-TKIs, as well as their significance in the course of the disease. According to longitudinal studies, certain driver co-alterations had already been detected in patients’ ctDNA samples before the EGFR-TKIs initiation, indicating the spatial tumor heterogeneity. Concerning patients who progressed on EGFR-TKIs, each line of treatment led to increased somatic genetic alterations in their ctDNA samples, reflecting the temporal tumor heterogeneity. It is worthy of note that lower genetic co-alterations were identified in responders’ ctDNA in comparison with nonresponders [104]. Consequently, liquid biopsies at baseline and progression may constitute a useful tool for identifying the individual intrinsic or emerging mechanisms of resistance to EGFR-TKIs and guiding the subsequent therapeutic decisions [14].

## 4. Discussion

EGFR mutant NSCLC is a constantly evolving field in contemporary oncology. Common and rare EGFR mutations may develop during the course of the disease affecting both the biological behavior of the tumor and the response to treatment. Currently, the TKIs represent the standard of care for EGFR mutant NSCLC. Third-generation TKIs have significant potential in patients who experience progression on other agents and particularly in those with CNS mutations [187,188]. Systemic progression on osimertinib remains challenging. While treatment schemes combining platinum-based chemotherapy with ICIs and/or anti-VEGF factors are recommended in this case, including the IMpower 150 regimen, developing more effective treatment strategies is a dire need [81,189]. 

Recent evidence has shown promising results regarding the combination of ICIs with chemotherapy in EGFR-T790M mutant NSCLC [190]. Nonetheless, the sequential systemic treatment that includes ICI and targeted agents should be carefully planned to reduce the risk of immune-related adverse events (irAEs). It has been documented that the sequential use of PD-(L)1 blockade and osimertinib is related to severe irAEs, especially pneumonitis and colitis. Interestingly, this phenomenon seems to be a drug-specific interaction between osimertinib and anti-PD(L)-1 agents, only when immunotherapy preceded osimertinib, not vice versa. Other EGFR-TKIs have not been reported to increase immune-related toxicity [191]. Additional combination treatment strategies, such as the combination of osimertinib and bevacizumab, are currently being evaluated based on their efficacy in CNS. According to a phase 1/2 clinical trial, their combination was both effective and tolerable, and an ongoing phase 3 clinical study will cast light on the magnitude of its clinical benefit (ClinicalTrials.gov, NCT04181060) [192]. The underlying mechanism of the aforementioned synergistic effects has yet to be unveiled. 

Liquid biopsies might contribute significantly to this end, enabling physicians to make more precise treatment decisions in response to systematic progression. While repeated liquid biopsies as a means of early detection of mutation loss, gene fusion or even transformation from NSCLC to SCLC appear promising [182], their sensitivity and specificity remain concerning, given that a negative liquid biopsy still needs to be validated with a conventional tissue biopsy. The capacity of Next Generation Sequencing (NGS) to reliably detect treatment-induced mutagenesis via liquid biopsy before and after targeted therapy with a positive predictive value of almost 89% [193] should be validated and expanded. Future research should also take into account the potential of dose escalation to increase the effectiveness of existing treatment—taking into account the example of osimertinib dose escalation in CNS metastases, the mutation profile of both primary and metastatic tumors as a means to predict the response to targeted agents and the co-existence of EGFR mutations with other mutations or molecular traits that may alter the response to treatment. The latter is quite important concerning EGFR mutant NSCLC treatment with ICIs because assessing the expression of PD-L1 molecules on tumor cells may help personalize immunotherapy and improve its outcomes. 

Furthermore, allosteric EGFR inhibitors emerge as a promising approach towards overcoming resistance to osimertinib. Given that they usually exhibit synergistic activity with osimertinib and their mechanism of action is independent of common ATP-binding site mutations, they might constitute the next-generation agents against EGFR mutant NSCLC. These innovative mutant-selective inhibitors were mainly designed to bind in a pocket adjacent to the ATP-binding site, instead of the ATP site itself, inducing the stabilization of the inactive “C-helix out” conformation of the kinase and, thus, its inhibition. This partially explains the major limitation of the early allosteric inhibitors, namely EAI001, EAI045, and DDC4002, that failed to demonstrate robust single-agent antitumor activity [194,195]. With the intent to thwart the allosteric pocket occlusion that is induced by the EGFR dimerization, the combination treatment of the EAI045 and the dimerization-disrupting anti-EGFR monoclonal antibody, cetuximab, was recently evaluated. Indeed, the combination showed antitumor activity against L858R/T790M and L858R/T790M/C797S EGFR mutant NSCLC cellular assays [196,197]. Therefore, emerging treatment strategies that encompass combinations ATP-competitive and allosteric inhibitors may enhance the efficacy of EGFR inhibition and reduce the accumulation of various resistant mutations, given that they target different binding pockets. On the other hand, new, more potent agents, such as JBJ-04-125-02 and JBJ-09-063, have already demonstrated single-agent activity against L858R/T790M and L858R/T790M/C797S mutations, indicating their higher affinity. Besides the efficacy, the synergistic effect of JBJ-04-125-02 and osimertinib might allow the reduction of the dosage of both agents and, thus, be beneficial to patients in terms of toxicity [194].

Additionally, future research should expand the landscape of mutations implicated in NSCLC development and progression. A growing body of research has emphasized the role of seven transmembrane G protein-coupled receptors (GPCRs) in various cancers, including lung cancer. In this context, GPCR 16 has been shown to decrease adhesion and increase cellular proliferation in NSCLC in the presence of two novel oncogenic proteins, namely endothelial transcription factor 2 (E2F2) and EGF-like module-containing, mucin-like, hormone receptor-like 2 (EMR2). Similarly, GPCR 124 has been shown to induce resistance to gefitinib, which can be countered by microRNA miR-138-5p expression. The role of these receptors, which interfere with multiple pathways of G-coupled cellular signaling has already been researched in lung adenocarcinoma [198,199,200]. It warrants further investigation in the context of NSCLC, where evidence regarding GPCRs stems mainly from studies focusing on different mutations or drug resistance mechanisms. Finally, yet importantly, adapting practice guidelines to the genomic epidemiology of EGFR at a regional level is a promising endeavor, which has recently been implemented in Asia [201].

## 5. Conclusions

In conclusion, TKIs resistance in EGFR mutant NSCLC constitutes a growing concern in oncology. A plentitude of EGFR-dependent and independent mutations have the potential to downplay the effect of numerous regimens, including the third-generation TKI, osimertinib. Subsequently, the PFS and the OS of patients are compromised. Digging deeper into the etiology of the disease and delving into personalized medicine can pave the way out of this stalemate. The field will benefit from consistent study methodologies to better map the landscape of mutations and their clinical implications. Further implementation of liquid biopsy in combination with tissue rebiopsy in selected patients is warranted as a means of monitoring the response to EGFR-TKIs and early detection of progression. Liquid biopsies may also facilitate the early detection of either intrinsic or acquired resistance and formulate the optimal therapeutic strategy. ADCs, bi-specific antibodies, pan-HER inhibitors, allosteric EGFR inhibitors and other tailored treatments have recently enriched the therapeutic arsenal against EGFR mutant NSCLC with intrinsic or acquired resistance to EGFR-TKIs. Ongoing molecular profiling studies concerning these patients form a prelude to a new era of individualized, precise and targeted therapy.

## Figures and Tables

**Table 2:** Studies evaluating TKIs in EGFR mutant NSCLC brain metastases—EGFR mutant NSCLC (EGFR mut); Progression-Free Survival (PFS); Response rate according to the version 1.1 of the Response Evaluation Criteria in Solid Tumors (RR); Overall control rate (ORR); Disease Control Rate (DRR); Duration of response (DoR); Overall survival (OS); Hazard ratio (HR), Blood–Brain Barrier (BBB).

Reference	TKIs	Study Design	Outcomes
Y.L. Wu et al., 2013 [144]	Erlotinib	Phase II Clinical Trial including 48 patients with EGFR mutant and non-EGFR mutant NSCLC BMs previously treated with first-line platinum-doublet chemotherapy	Median PFS: 10.1 months; EGFRmut median PFS: 15.2 months; EGFR wt median PFS: 4.4 months
Schuler et al., 2016 [77]	Afatinib or cisplatin plus pemetrexed	Clinical trial recruiting patients with metastatic EGFR mutant NSCLC; subgroup analysis of patients with brain metastases	Median PFS with afatinib: 8.2 months; Median PFS with chemotherapy: 5.4 months
Ballard et al., 2016 [145]	Osimertinib	Preclinical assessment of Osimertinib CNS penetration in animal models	Osimertinib was superior to gefitinib, rociletinib (CO-1686), or afatinib in terms of penetration of the mouse BBB, Osimertinib induced sustained tumor regression in an EGFRmut PC9 mouse brain metastases model, where rociletinib failed
J.C.-H. Yang et al., 2017 [146]	Osimertinib	AURA—Phase I/II Clinical trial involving 201 patients with asymptomatic, stable T970M+ brain metastases that did not require corticosteroids	ORR: 62%; DRR: 90%; Median PFS: 12.3 months
Arbour et al., 2018 [147]	Erlotinib (pulse/continuous-dose erlotinib)	Phase 1 clinical trial with 19 patients with EGFR mutant NSCLC brain metastases	RR in brain metastases: 74%; overall median PFS: 10 months
Y.-L. Wu et al., 2018 [148]	Osimertinib	Randomized Phase III Trial (AURA3)—analysis reporting the CNS effectiveness of osimertinib versus platinum-pemetrexed chemotherapy in patients with EGFR T790M+ advanced NSCLC who experience disease progression with prior EGFR-TKI treatment	CNS ORR in patients with ≥1 measurable CNS lesions: 70% with osimertinib and 31% with chemotherapy; median CNS PFS: 11.7 months with osimertinib and 5.6 months with chemotherapy
J.C.H. Yang et al., 2020 [149]	Osimertinib 160 mg	Phase I clinical trial BLOOM; 41 patients with leptomeningeal metastases from EGFRmut advanced NSCLC with a history of disease progression on previous EGFR-TKI therapy	ORR: 41%; median DoR: 8.3 months; median PFS: 8.6 months; median OS: 11.0 months; safety and toxicity consistent with previous knowledge
Park et al., 2020 [150]	Osimertinib	Phase II, multicentre, two cohort study of 160 mg osimertinib in EGFR T790M+ NSCLC patients with brain or leptomeningeal metastases and a history of progression on previous EGFR TKI therapy	Median PFS: 7.6 months; Median OS: 16.9 months, Previous radiotherapy favored increased PFS (HR: 0.42)
Piper-Vallillo et al., 2020 [151]	Osimertinib	Retrospective real-world cohort of EGFRmut NSCLC patients with brain or leptomeningeal metastases on osimertinib 80 mg, dose escalation to 160 mg	Dose escalation increased PFS by 3.6 months and improved CNS disease control
H. Wang et al., 2021 [152]	1st generation EGFR TKIs alone or combined with chemotherapy or bevacizumab	Retrospective analysis of 584 EGFRmut advanced NSCLC patients	1st generation EGFR TKIs plus bevacizumab achieved the highest intracranial PFS (27.2 months), 1st generation EGFR TKIs alone achieved the highest OS (27.8 months)—no available data for the same on 1st generation TKIs plus bevacizumab
